# A Low-Dose Combination of Fluvastatin and Valsartan: A New “Drug” and a New Approach for Decreasing the Arterial Age

**DOI:** 10.1155/2015/235709

**Published:** 2015-03-03

**Authors:** Miodrag Janić, Mojca Lunder, Mišo Šabovič

**Affiliations:** Department of Vascular Diseases, University of Ljubljana Medical Centre, Zaloška Cesta 7, 1000 Ljubljana, Slovenia

## Abstract

We have developed a new “drug” and approach that appear to be effective in reducing arterial age. This “drug” represents a low, subtherapeutic dose of statin and sartan and particularly their low-dose combination. The improvement of arterial wall characteristics, also reflecting in a decrease of arterial age, was achieved after a short period of treatment (one month) with the above-mentioned drugs. In addition, we have also implemented a new, innovative therapeutic approach, consisting of intermittent (cyclic) treatment—alternating short “treatment” periods and much longer “rest” periods (when the beneficial effects are still present but gradually decline). This new “drug” and approach both merit further investigation in order to confirm their antiaging efficacy.

## 1. Introduction

Arterial aging is a process that occurs as part of whole body aging. The role, contribution, and importance of arterial aging to whole body aging have not yet been clarified. Nevertheless, it seems logical that arterial aging should have an important, if not pivotal, role in the aging of the whole body. Therefore, arterial aging could be a valuable target for antiaging interventions. Furthermore, arterial aging also substantially contributes to cardiovascular disorders and represents an important risk factor. It is also of note that a person's age is reflected in their arterial aging.

Although significant advances have been achieved in the prevention and treatment of cardiovascular diseases, they still remain the most important factor of morbidity and mortality in the developed world [1]. Consequently, new strategies for their more effective prevention are desirable [2], as are strategies to allow successful aging. Thus, at least in theory, slowing or even reversing arterial aging could result in both antiaging and cardiovascular preventive effects and benefits. We have previously described and introduced a new, innovative preventive approach, which we have now explored even further. In contrast to other implemented approaches, our concept targets the arterial wall directly rather than the risk factors for aging and atherosclerosis. Notably, the treatment of arterial aging has not been described as an antiaging approach.

We therefore propose a new “drug(s)”: low, subtherapeutic doses of statins and sartans and particularly their combination. In addition, we propose a new innovative approach. The approach consists of “intermittent” treatment, that is, one-month therapy followed by a 6–12 month free-of-treatment “rest” period (when the beneficial effects are still present but gradually decline). This period is then followed by a new treatment cycle [3]. The efficacy of the described approach has been studied on apparently healthy male individuals [4–6], as well as patients with diabetes mellitus type 1 [7] and type 2 and patients surviving myocardial infarction. Herein, we describe and present our approach in detail, combining data from the spectrum of our different studies.

## 2. Arterial Age

Age is an important risk factor for cardiovascular events and cardiovascular risk calculators are based on age, such as the most widely used Framingham Risk Score and SCORE [8]. With regard to age, some studies suggest that arterial (vascular) age should be considered in risk prediction models instead of chronological age, especially for young or middle-age people with low cardiovascular risk [9–11]. Thus, arterial (vascular) age can be defined as an individual's age after considering their functional and structural arterial wall properties and represents the age at which an individual's arterial wall parameter level would be in the healthy population mean [11]. Importantly, it should be considered that chronological and arterial (vascular) age are not always strictly parallel. Moreover, it is much more common that both “types” of aging continue at a different pace. From a clinical point of view, arterial age seems to be more important as a risk and prognostic factor for cardiovascular events, since it more reliably reflects the real age of an individual than his or her chronological age.

For an individual, the concept of arterial age is more understandable than cardiovascular disease risk [10–12]. Several methods exist for calculating arterial age, some of which are based on coronary calcium score determination [13], such as the MESA (Multi-Ethnic Study in Atherosclerosis) arterial age calculator [14]. In another study, arterial age calculation was based on nomograms of carotid intima-media thickness (cIMT) [9]. Arterial age calculation can be based directly on the structural and functional arterial wall parameters, such as in pulse wave velocity (PWV) and cIMT [11]. The importance of arterial aging has been confirmed by calculations of vascular aging from SCORE and Framingham scores that have recently been extended for arterial age determination and calculations [10, 15].

We believe that arterial age is a very important factor in the aging process, since arteries are the body's main system that interconnects all body organs. Consequently, arterial age could be a surrogate for biological age. If we can achieve arterial age decrease, we can logically expect an improvement of the function of other body systems, resulting in a decrease in the whole body's age. Thus, arterial age could be an ideal target for any antiaging intervention. Decreasing arterial age could result in double benefits: a decrease in arterial age and whole body age and a decrease in cardiovascular risk. Research targets should therefore be focused on arterial age in order to try to obtain efficacious treatment for arterial health prevention in the near future. Taken all together, the rationale is to reduce arterial age. Both age and cardiovascular risk decrease would be possible by targeting the arterial age. In this regard, a new “drug” and approach, which would effectively target arterial age, seem to be a straightforward solution in antiaging medicine.

## 3. Innovative “Drug”

We propose a new “drug,” which, at least in theory, could be beneficial in reducing injury of the arterial wall induced by aging. It consists of two widely used drug groups with known beneficial effects on the arterial wall: statins and sartans or their combination. These beneficial effects are so-called “pleiotropic effects” and are beyond their primary mode of action, namely, plasma cholesterol reduction and blood pressure lowering. Beneficial pleiotropic effects are manifested through various mechanisms, the most important being those that are similar in both drug groups: oxidative stress reduction, inflammation lowering, and other potential mechanisms, exerted directly on the arterial wall [16–18]. Due to the described mechanisms, these two drug groups represent a fairly logical choice for arterial antiaging, as the aging process is the consequence of their perpetual activation. Their efficacy on arterial function improvement has also been explored, and they have been proved to be efficient to a certain extent in therapeutic doses. At therapeutic doses, their effect could be purely the result of their primary mode of action [19–22]. It is very important to consider that the pleiotropic effects of the drugs described appear even when they are administered at low doses, that is, doses that do not exert their primary effect on cholesterol or blood pressure [23, 24]. This phenomenon is of paramount importance for our approach. In addition, in subtherapeutic doses, these drugs have almost no side effects and are particularly safe [25, 26].

As mentioned above, pleiotropic effects are not strictly dose-dependent as they do not always appear in parallel with therapeutic effects. For example, if a higher dose of a certain statin reduces cholesterol to a higher degree, this does not mean that its pleiotropic effects are also greater at a higher dose. Thus differences appear in the pleiotropic effects of low and high doses, particularly in the pleiotropic effects on the arterial wall. For example, rosuvastatin was shown to possess more beneficial pleiotropic effects at low doses than at high doses. In low doses, rosuvastatin significantly increased capillary density and accelerated blood flow in a mouse model of surgically induced limb ischemia. This was accompanied by an increase in circulating endothelial progenitor cells, dependent on endothelial NO-synthase activation. These effects were not observed with rosuvastatin at high doses [27].

**Figure 1:**
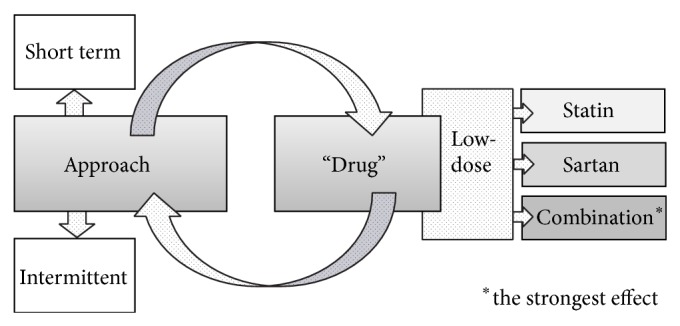
Schematic presentation of the “drug” and approach. The approach is formed of a short-term treatment period, followed by much longer period without treatment, that is, intermittent approach. The “drug” consists of two widely used drug groups with known beneficial effects on the arterial wall: low-dose statins and sartans or their low-dose combination.

Based on the aforementioned assumptions, we proposed a new approach to improving arterial wall characteristics, choosing statin, sartan, or their combination at low doses. Low doses were selected in order to achieve maximal pleiotropic beneficial effects on the arterial wall and to avoid any potential side effects. We chose low-dose fluvastatin, valsartan, or their low-dose combination for our clinical studies. We found that already after a short period (14 days to 1 month) of treatment with a low-dose combination of fluvastatin and valsartan or separate drugs, the functional and structural arterial wall characteristics improved; that is, PWV and beta-stiffness were reduced and flow-mediated dilation (FMD) improved (Figure 1). These results confirm the strong efficacy of such a low-dose drug combination. At the low doses used, the drugs do not exhibit their primary mode of action [3]. These facts were confirmed in our previous studies, along with the fact that combination treatment was superior to separate drugs [4–6]. This effect could be additive or even synergistic [28–31]. Notably, since this kind of low-dose combination has not been investigated previously, it represents an innovative “drug.”

## 4. Innovative Approach

We introduced intermittent or cyclic treatment, where a cycle means a short period of treatment and a long period without treatment. We believe that intermittent treatment is more effective, since it does not allow rebound mechanisms to develop that could be particularly important for the inhibition of the pleiotropic effects. It is worthy of mention that in animal studies we observed that, after reaching a peak, the effects decline with the continuation of the same treatment; this is probably due to the reasons described above [32]. Additionally, better compliance and almost no side effects are achieved by short-term treatment.

**Figure 2:**
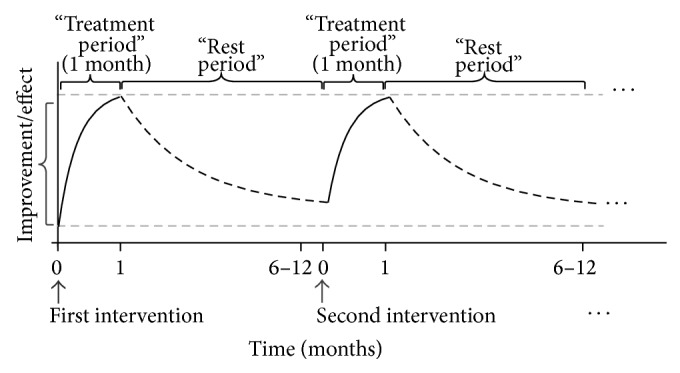
A scheme representing the intermittent approach. The approach consists of a short (one-month) treatment period followed by a relatively much longer rest period (from a few to 12 months). During the rest period, the beneficial effects are still present but gradually decline.

We have tested this intermittent cycling approach in several studies. The approach consists of a short (one-month) treatment period followed by a relatively much longer rest period (from a few to 12 months). During the rest period, the beneficial effects are still present but gradually decline (Figure 2). We observed that the so-called “rest” period should be shorter in participants with already injured arterial wall (patients with diabetes mellitus, after myocardial infarction) and longer in healthy persons [7]. This observation is quite logical and supports our idea.

## 5. Improvement of Arterial Wall Characteristics

The drug combination and therapeutic approach described above specifically target the arterial wall. The latter has two types of characteristics: functional, represented by endothelial function, and structural, represented by the arterial stiffness. However, arterial stiffness is more complex, as it is influenced by functional and structural changes.

The functional characteristics of the arterial stiffness are regulated by the vascular smooth muscle cells (VSMC), which are in turn regulated by vasoactive hormones, the most important one being nitric oxide (NO), released by the endothelium. These characteristics might therefore be improved through improvement of the endothelium. For this kind of improvement, the effects can vary from immediate to long-term, but on the other hand, for structural characteristics improvement, a longer time is needed to allow for remodeling. The structural characteristics are defined by the ratio between collagen and elastin in the arterial media, as well as the amount of viable VSMC that produce these compounds. The higher the amount of elastin, the more elastic the artery, while, on the other hand, the artery becomes stiffer with age, when collagen predominates and becomes cross-linked. This particular characteristic is also influenced by the functional characteristics of the arterial wall. For arterial stiffness improvement/decrease, two possible approaches could be used: (i) decrease in functional and (ii) decrease in structural effects on arterial stiffness. Our approach interferes with and influences both.

A one-month treatment with a low-dose combination of fluvastatin and valsartan improves the functional arterial wall characteristics. Through these and additional mechanisms, it also subsequently influences the structural arterial wall characteristics, leading to overall beneficial effects on the arterial function. The improvement in functional characteristics is evident in an increase in FMD, while the structural characteristics' improvement is evidenced by a decrease in both PWV and *β*-stiffness [4–6]. In addition to these observations, we have shown that these effects can at least in part be explained by a reduction in inflammation and oxidative stress [33].

## 6. Decrease of Arterial Age

We have proposed the following concept—if arterial stiffness increases with age, a decrease in arterial stiffness will result in decreasing arterial age. The decrease of arterial age was achieved by our approach in all studied groups of apparently healthy participants. After one month of treatment, low-dose fluvastatin or low-dose valsartan separately decreased arterial age by approximately 7 years, whereas a low-dose combination decreased arterial age by approximately 10 years. These calculations are based on nomograms for PWV and *β*-stiffness [34, 35].

## 7. Proof of the Concept

Aging influences the arterial wall through two basic interconnected mechanisms: structural and dynamic. Structural aging is the consequence of a higher collagen to elastin fibers ratio, leading to stiffer arteries. Dynamic aging is the consequence of reduced nitric oxide (NO) bioavailability, leading to endothelial dysfunction and a higher tone of smooth muscle cells in the arterial media. All these derangements have a similar pathophysiological background, namely, increased oxidative stress and inflammation in the arterial wall [36]. Additional, not yet fully discovered mechanisms probably also play a role. Nevertheless, all of the described derangements could be influenced, amongst other, also by the new “drug” and approach [33, 36].

In our studies, we have thoroughly tested the described new, innovative “drug” and approach. In apparently healthy middle-aged men, we tested the effect of one-month therapy with low-dose fluvastatin, valsartan, or their low-dose combination. We have consistently obtained improvement of arterial wall characteristics in every participant. These results were all statistically significant to a very high degree. In participants treated only with low-dose fluvastatin, PWV decreased by 6% and *β*-stiffness decreased by 11%, while FMD improved by 92%, all compared to the baseline values [4]. Low-dose valsartan showed slightly better results, with PWV decreasing by 8% and *β*-stiffness by 12% and FMD improving by 150% [5]. As expected, the low-dose combination of fluvastatin and valsartan proved to be the most potent. In the latter group of participants, PWV decreased by 11% and *β*-stiffness by 12% and FMD improved by 170% [6]. These results were further supported by an accompanying significant decrease in inflammatory markers, namely, high sensitivity C-reactive protein (hsCRP), vascular cell adhesion molecule-1 (VCAM-1), and interleukin 6 (IL-6) in the low-dose combination group. Additionally, oxidative stress parameters also significantly changed only in the low-dose combination group, with total antioxidant status (TAS) and glutathione peroxidase (GPx) increasing and selenium levels decreasing [33]. These molecular results partially elucidate the mechanism behind the approach. Furthermore, the effectiveness of a 30-day treatment with a low-dose combination of fluvastatin and valsartan was also proved in patients with diabetes mellitus type 1; PWV decreased by 7.5% and *β*-stiffness decreased by 10% while FMD improved by 73% [7]. Importantly, we have observed no side effects in any of our study participants. This was somehow expected, as low, subtherapeutic doses of statins and sartans were used, thus not producing any blood pressure or lipids reduction.

When the arterial wall parameters were monitored after discontinuation of the one-month treatment, the protective pleiotropic effects were still present over a certain period of time but gradually declined. The decline was the fastest in the low-dose fluvastatin group, followed by the low-dose valsartan group [4, 5]. In the low-dose combination group, the residual effect five months after treatment discontinuation was somehow surprisingly still at almost 80% and was halved only after 10 months (therefore not yet reaching the basal level) [6], thus allowing for a probable one-year cycle period. Our preliminary observations show that after these effects reached the basal level, repeating a one-month treatment with the same drug/combination as the previous time gave the same results as the first treatment. When these results are taken together, a constant cyclical achievement of the basal level results or basal level arterial age could be achieved with the described intermittent approach over several years or even longer. Evidently, the arterial aging process can be slowed down. Furthermore, on the one hand, such treatment leads to obtaining or regaining some “arterial” years that would be otherwise lost and would lead to a more rapid decline in arterial function, preclinical atherosclerotic changes, and later on to atherosclerotic manifestation. On the other hand, such treatment leads to the slowing of whole body aging.

This concept was proven even further in animal studies with isolated rat heart and aorta, where we obtained the highest increase in endothelium-dependent thoracic aorta relaxation and coronary flow after six weeks of treatment with low-dose statin, sartan, or their low-dose combination. As in human studies, the low-dose combination was the most effective. It should be emphasized that, after eight weeks of treatment, the described protective effects declined, thus proving the assumption on the activation of counter compensatory mechanisms [32]. Therefore, an optimal length of therapy should be used in order to obtain the maximal possible effect on the one hand and avoid triggering the compensatory mechanisms that diminish protective effects on the other. The results in animal studies were further backed by gene expression experiments, where the expression of endothelin receptor type A (EDNRA) gene was reduced, while the expression of endothelial nitric oxide synthase 3 (NOS3) gene increased [37].

## 8. Future Perspectives

We have shown that our new “drug” and approach are effective in a varied range of participants, from apparently healthy to unhealthy ones. We believe that ideal candidates for treatment would be middle-aged males and females, still healthy or with existing cardiovascular disease, diabetes mellitus, or overt atherosclerotic disease, and also the elderly. We believe that the new “drug” and approach could be somehow effective in these people as well, despite their different characteristics and the drugs they already use.

All in all, our previous studies have confirmed the effectiveness of our new innovative “drug” and approach to improving arterial wall properties and consequently decreasing arterial age. We propose that repetition of treatment cycles (alternating treatment and rest periods) through the years could preserve arterial age at approximately the same level for a longer period of time. Obviously, several additional studies are necessary to confirm the effectiveness of our preventive approach. Further animal and human studies exploring the exact mechanisms underlying the observed beneficial effect are also required. Larger and long-lasting studies are needed in order to test whether this approach influences whole body aging and reduces cardiovascular events in the long term.
